# Impact of Cyclic Error on Absolute Distance Measurement Based on Optical Frequency Combs

**DOI:** 10.3390/s24113497

**Published:** 2024-05-29

**Authors:** Runmin Li, Haochen Tian, Junkai Shi, Rongyi Ji, Dengfeng Dong, Weihu Zhou

**Affiliations:** 1 Division of Time and Frequency Metrology, National Institute of Metrology, Beijing 100029, China; 2 Optoelectronic Technology Center, Institute of Microelectronics of the Chinese Academy of Sciences, Beijing 100029, China; 3 Key Laboratory of State Administration for Market Regulation (Time Frequency and Gravity Primary Standard), Beijing 100029, China; 4 University of Chinese Academy of Sciences, Beijing 100049, China

**Keywords:** absolute distance measurement, cyclic error, synthetic wavelength interferometry, single-wavelength interferometry

## Abstract

Absolute distance measurements based on optical frequency combs (OFCs) have greatly promoted advances in both science and technology, owing to the high precision, large non-ambiguity range (NAR), and a high update rate. However, cyclic error, which is extremely difficult to eliminate, reduces the linearity of measurement results. In this study, we quantitatively investigated the impact of cyclic error on absolute distance measurement using OFCs based on two types of interferometry: synthetic wavelength interferometry and single-wavelength interferometry. The numerical calculations indicate that selecting a suitable reference path length can minimize the impact of cyclic error when combining the two types of interferometry. Recommendations for selecting an appropriate synthetic wavelength to address the tradeoff between achieving a large NAR and minimizing the risk of failure when combining the two methods are provided. The results of this study are applicable not only in absolute distance measurements but also in other applications based on OFCs, such as surface profile, vibration analysis, etc.

## 1. Introduction

Absolute distance measurements with nanometer precision have significantly propelled advancements in both science and technology, such as the monitoring of large facilities in big science, manufacturing of nanotechnology-based products, satellite formation flying, etc. [1,2,3,4]. Single-wavelength interferometry is one of the most important and well-developed methods for realizing high-precision distance measurements because the optical wavelength is the measuring benchmark [5]. However, the non-ambiguity range (NAR) of single-wavelength interferometry is half the optical wavelength, which is typically several hundreds of nanometers. Therefore, single-wavelength interferometry is used for incremental measurements. To realize absolute distance measurement with nanometer resolution, researchers usually combine single-wavelength interferometry with other methods that offer a larger NAR, such as synthetic wavelength interferometry [6,7,8], dispersive interferometry [9,10,11,12,13], and time-of-flight measurement [3,14,15,16,17,18]. The dispersive interferometry offers a high update rate and a high resolution but suffers from the trade-offs in NAR and resolution. Time-of-flight measurement based on dual-comb lasers has the advantage of large NAR and a high update rate. However, the high power penalty, complex configuration and inherent trade-offs in update rate, NAR, and resolution of this method limit its applications. Synthetic wavelength interferometry has a configuration similar to that of single-wavelength interferometry, which allows for the combination of two types of interferometry on one platform. Meanwhile, the tuning flexibility of the synthetic wavelength enables cascaded NAR extensions. The generation of a stable synthetic wavelength, which is the key point for synthetic wavelength interferometry, relies on either two phase-locked continuous wave (CW) lasers or an optical frequency comb (OFC) [6,7,8,19,20,21,22,23]. The sophisticated phase-locking systems for stabilizing the CW lasers make the laser source complex and costly. An optical frequency comb, which contains numerous frequency-stabilized comb modes, is served as the ideal laser source for performing synthetic wavelength interferometry. By selecting different comb modes pair, stable synthetic wavelength ranges from several tens of micrometers to several meters is obtainable, enabling precise absolute distance measurement over long distances.

In interferometry-based distance measurements using OFC, cyclic error is one of the common phenomena that causes uncertainty. The cyclic error exhibits periodic deviation from the true value, which is attributed to the disturbance of crosstalk signals during phase measurement [24,25]. Possible reasons that may induce crosstalk signals include optical crosstalk signals, such as polarization crosstalk and spurious reflection, and electrical crosstalk signals, such as electromagnetic contamination and so on [25,26,27]. Various methods have been proposed to reduce cyclic error in response to the different origins, including polarization management, phase compensation, careful design of electrical circuits, etc. [28,29,30,31,32,33,34,35,36]. Data processing and some algorithms are useful for compensating cyclic error [36,37,38,39,40]. H. Kang et al. demonstrated a cyclic-error-free system by dynamically linking the synthetic wavelength to a divisor of the distance under test [41]. However, eliminating all the crosstalk signals from one system is extremely difficult. More importantly, cyclic error in synthetic wavelength interferometry may lead to mistakes in calculating the integer order of single-wavelength interferometry, resulting in the failure to combine the two types of interferometry. Such mistakes severely deteriorate the accuracy of one system. Therefore, a quantitative investigation of the cyclic error and its impact on the combination of synthetic wavelength and single-wavelength interferometry is essential for the analysis and optimization of system performance.

In this study, we quantitatively investigate the influence of cyclic error on heterodyne interferometry-based distance measurement using an OFC. Simulations are conducted to analyze the probability of correctly retrieving integer information of single-wavelength interferometry using synthetic wavelength interferometry. The investigation considered various parameters, such as the phase and intensity of crosstalk, reference path length offset, and different ratios between synthetic wavelengths and single wavelengths. According to our numerical simulation, the impact of cyclic error on the integer information of precise measurement can be minimized by slightly adjusting the reference path length. The careful selection of an appropriate synthetic wavelength is essential and advantageous for reducing the probability of incorrect integers.

## 2. Principle

**Figure 1:**
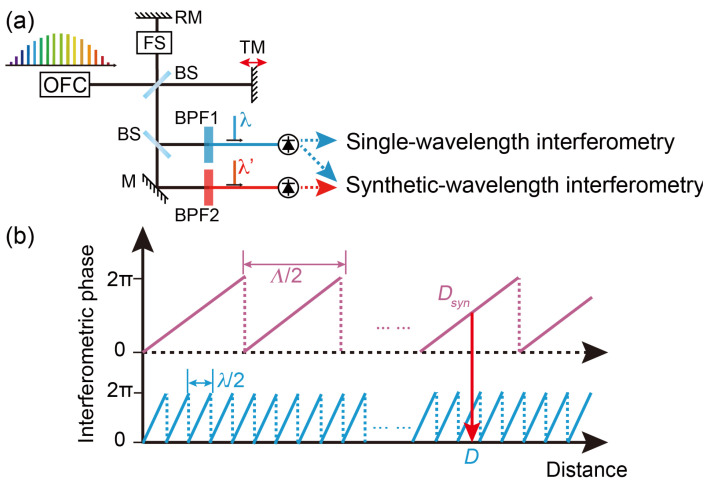
(**a**) General configuration of heterodyne-interferometry-based distance measurement. OFC: optical frequency comb; BS: beam splitter; FS: frequency shifter; DM: dichroic mirror; RM: reference mirror; TM: target mirror; M: mirror; BPF: bandpass filter. (**b**) Absolute distance measurement combining synthetic wavelength interferometry and single-wavelength interferometry.

The general setup of the distance measurement based on heterodyne interferometry is shown in Figure 1. The OFC is split into reference and target beams. The reference beam is frequency-shifted and reflected by a fixed reference mirror. The target beam is combined with the reference beam after being reflected by a moving target mirror. The recombined beam is detected by two photodetectors after passing through a beam splitter and two bandpass filters with center wavelengths of *λ* and *λ*′, respectively. Two heterodyne beats are detected by the photodetectors. For single-wavelength interferometry, the distance under test, which is the path length difference between the reference path and target path, is obtained by the phase of one heterodyne signal through *D* = *D_tar_* − *D_ref_* = *φλ*/4π*n* + *N* × *λ*/2*n*. Here, *φ* is the phase of the heterodyne signal, *n* is the air refractive index, *N* is an integer, and *D_tar_* and *D_ref_* are the target and reference path lengths, respectively. The NAR of single-wavelength interferometry is *λ*/2*n*. For synthetic wavelength interferometry, the measured distance is *D_syn_* = *ΦΛ*/4π*n* + *M* × *Λ*/2*n*, where *Λ* = *λλ*′/(*λ* − *λ*′) is the synthetic wavelength, *Φ* = *φ* − *φ*′ is the phase discrepancy between two heterodyne beats, *φ*′ is the phase of the heterodyne signal at *λ*′ and *M* is an integer. The NAR of synthetic wavelength interferometry is *Λ*/2*n*. By combining synthetic wavelength interferometry and single-wavelength interferometry, the integer *N* is determined as *N* = INT(*D_syn_*/(*λ*/2*n*)), where INT(·) denotes the operation of the rounded-off numbers. Consequently, distance measurement with sub-wavelength measurement resolution in the extended NAR is achieved, as shown in Figure 1b.

To realize distance measurements with high precision, the key is to detect the interferometric phase with high accuracy. Assuming that the phase of the heterodyne signal is detected by a lock-in amplifier, the tangent of the retrieved phase in an ideal case is:

(1) $$\tan\left(\varphi_{ideal}\right)=\frac{\mathrm{LPF}\left(V_{s}\left(t\right)\times V_{rI}\left(t\right)\right)}{\mathrm{LPF}\left(V_{s}\left(t\right)\times V_{rQ}\left(t\right)\right)}=\frac{A_{s}\sin\left(\varphi_{r}-\varphi_{s}\right)}{A_{s}\cos\left(\varphi_{r}-\varphi_{s}\right)}.$$

where *V_s_*(*t*) = *A_s_* × sin(2π*f_m_t* + *φ_s_*) is heterodyne signal; *f_m_* is twice the shifted frequency; *A_s_* is its amplitude and *φ_s_* = 4π*nD*/*λ* is its phase; *V_rI_*(*t*) = *A_r_* × sin(2π*f_m_t* + *φ_r_*) and *V_rQ_*(*t*) = *A_r_* × cos(2π*f_m_t* + *φ_r_*) are the in-phase and quadrature components of a reference signal, respectively; *A_r_* is its amplitude; *φ_r_* is its phase; and LPF(·) means removing high frequency components through low pass filter. However, in the real world, the measured phase of heterodyne signals often deviates from the ideal case because of disturbances that are induced by weak crosstalk signals. Assuming that the crosstalk signal is *V_n_*(*t*) = *A_n_* × sin(2π*f_m_t* + *φ_n_*) with an amplitude of *A_n_* and phase *φ_n_*, the detected signal becomes *V*(*t*) = *V_s_*(*t*) + *V_n_*(*t*). Therefore, the tangent of the retrieved phase with crosstalk is:

(2) $$\tan\left(\varphi_{real}\right)=\frac{\mathrm{LPF}\left(V\left(t\right)\times V_{rI}\left(t\right)\right)}{\mathrm{LPF}\left(V\left(t\right)\times V_{rQ}\left(t\right)\right)}=\frac{A_{s}\sin\left(\varphi_{r}-\varphi_{s}\right)+A_{n}\sin\left(\varphi_{r}-\varphi_{n}\right)}{A_{s}\cos\left(\varphi_{r}-\varphi_{s}\right)+A_{n}\cos\left(\varphi_{r}-\varphi_{n}\right)}.$$

The difference between Equations (1) and (2) indicates a periodic deviation between the retrieved phase with and without crosstalk, which is:

(3) $$\tan\left(\varphi_{real}\right)-\tan\left(\varphi_{ideal}\right)=\frac{A_{s}\times A_{n}\sin\left(\varphi_{s}-\varphi_{n}\right)}{A_{s}^{2}\cos^{2}\left(\varphi_{r}-\varphi_{s}\right)+A_{n}\times A_{s}\cos\left(\varphi_{r}-\varphi_{n}\right)\times \cos\left(\varphi_{r}-\varphi_{s}\right)}.$$

where *φ_s_* − *φ_n_* = 4π*nD*/*λ* − *φ_n_* = 4π*nD_tar_*/*λ* − *φ_c_* and *φ_c_* = 4π*nD_ref_*/*λ* + *φ_n_* is defined as the phase of cyclic error.

This periodic deviation leads to cyclic error in single-wavelength interferometry. According to Equation (3), the amplitude of the deviation is related to the amplitude of the crosstalk signal. Its phase is determined by the phase of the crosstalk signal and the position of the reference mirror. 

**Figure 2:**
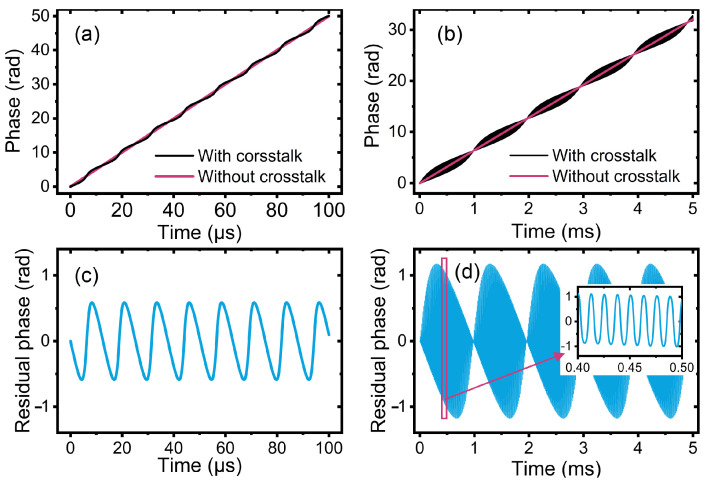
(**a**,**b**) Cyclic error in single-wavelength interferometry; (**c**,**d**) cyclic error in synthetic wavelength interferometry. Black and magenta curves represent the retrieved phases with and without crosstalk, respectively. Blue curve represents the phase difference between the black and magenta curves.

Assuming that the power of the crosstalk signal is 10 dB weaker than that of the heterodyne signal, the retrieved phases from single-wavelength interferometry with and without crosstalk are displayed as black and magenta curves shown in Figure 2a. The differences between them are shown in Figure 2b. The period of the cyclic error is *D* × mod(*λ*/2*n*), where mod(·) represents the modulo operation.

Considering that the interferometric phase of synthetic wavelength interferometry is obtained from the phase discrepancy between the two heterodyne signals, the cyclic errors in the two single-wavelength interferometry both affect the results of synthetic wavelength interferometry. Owing to the difference in the optical wavelengths, the cyclic errors in the two single-wavelength interferometry exhibited slightly different periods. Specifically, the beat between these two cyclic errors leads to the cyclic error in synthetic wavelength interferometry. Figure 2c,d show the retrieved phase of the synthetic wavelength interferometry and its cyclic error when the synthetic wavelength is 78 times the optical wavelength. The power of the crosstalk signals is 10 dB lower than that of the heterodyne signals. The amplitude of the envelope of the residual phase in synthetic wavelength interferometry is twice that of the residual phase, as shown in Figure 2d. Converting to distance, the cyclic error in synthetic wavelength interferometry is 2 × *Λ*/*λ* times that in single-wavelength interferometry. From the inset figure shown in Figure 2d, the period of the carrier is the average of the periods of the cyclic errors in the two single-wavelength interferometry.

**Figure 3:**
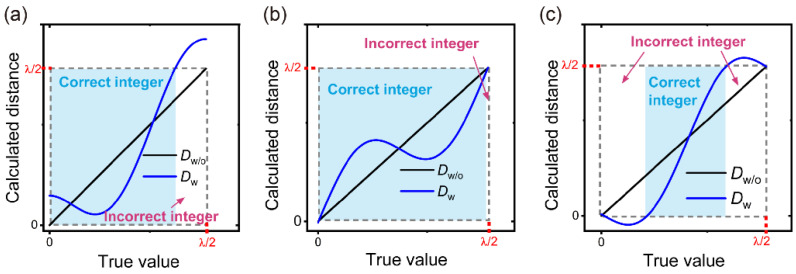
The impact of cyclic error in synthetic wavelength interferometry on determining the integer order of single-wavelength interferometry. The blue and black curves correspond to the calculated distance from synthetic wavelength interferometry with (*D_w_*) and without (*D_w_*_/*o*_) crosstalk, respectively. Blue box indicates the areas where the integer order could be correctly obtained. The phases of cyclic error are π/2, 0, and π in (**a**–**c**), respectively.

Because of the cyclic error in synthetic wavelength interferometry, the integer *N* may not be correctly determined using synthetic wavelength interferometry, as illustrated by Figure 3. As shown in Figure 3a, the phase of the cyclic error is set to π/2. The power of the crosstalk is 37 dB below that of the heterodyne signal. The synthetic wavelength is 78 times the optical wavelength. The horizontal and vertical axes represent the true value and calculated distances, respectively. The side length of the gray square is equal to the NAR of single-wavelength interferometry. The blue and black curves correspond to the calculated distance from synthetic wavelength interferometry with (*D_w_*) and without (*D_w_*_/*o*_) crosstalk, respectively. When *D_w_* falls within the gray square, the integer *N* can be correctly retrieved, as highlighted by the blue shadow. By contrast, the retrieved integer is incorrect when *D_w_* is beyond the gray square. Therefore, the ratio between the areas of the blue shadow and gray square indicates how well the integer information can be retrieved correctly. This ratio varies with the phase and intensity of the cyclic error. Figure 3b,c show the results when the phase of cyclic error is 0 and π, respectively.

## 3. Numerical Simulation and Discussion

**Figure 4:**
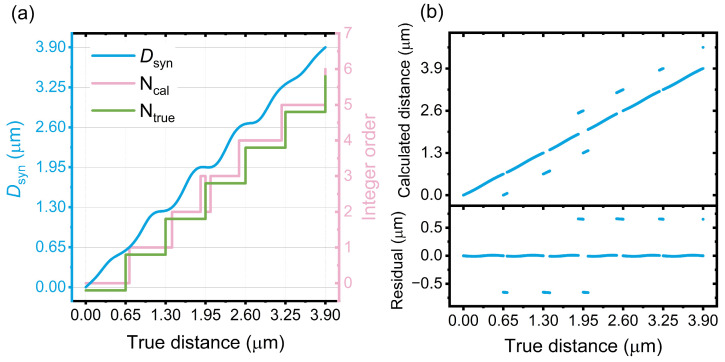
Numerical calculation of distance under test when the phase of cyclic error is 0. (**a**) Left axis: calculated *D_syn_* versus true distance (blue curve). Right axis: the integer order of single-wavelength interferometry calculated from *D_syn_* (*N_cal_*, pink curve) and its true value (*N_true_*, green curve). The green curve is shifted downward for clarity. (**b**) Comparative results between calculated distance and true distance. The discrete data points are due to incorrect integers.

**Figure 5:**
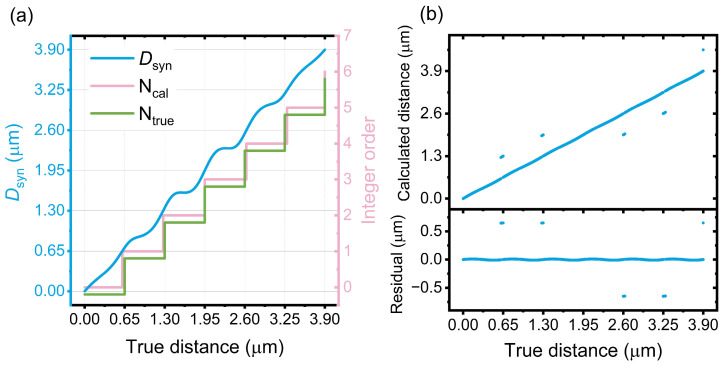
Numerical calculation of distance under test when the phase of cyclic error is π. (**a**) Left axis: Calculated *D_syn_* versus true distance (blue curve). Right axis: the integer order of single-wavelength interferometry calculated from *D_syn_* (*N_cal_*, pink curve) and its true value (*N_true_*, green curve). The green curve is shifted downward for clarity. (**b**) Comparative results between calculated distance and true distance. The discrete data points are owing to the incorrect integers.

In this section, quantitative simulations about the impact of cyclic error on distance measurement are performed. First, we choose the optical wavelengths of 1300 nm and 1560 nm, which can be obtained from a broadband OFC or a dual-color electro-optic frequency comb [42,43]. A synthetic wavelength of 7.8 µm is obtained for the simulation. In this case, the cyclic error of synthetic wavelength interferometry contains only a few carriers beneath one period of the envelope. The impact of the cyclic error on the estimation of *N* could be displayed with better clarification in Figure 4 and Figure 5, especially when the distance is around multiple times of NAR of single-wavelength interferometry. However, a longer synthetic wavelength is preferred for the purpose of extending NAR in practical cases. The power of the heterodyne signal is 0 dBm, whereas the power of crosstalk is −20 dBm. The displacement is set to 3.9 µm, which is equal to the NAR of synthetic wavelength interferometry. This enables a comprehensive analysis of the impact of cyclic error since the behavior of the measurements within the subsequent NAR is the same as that of the first NAR. When the true distance increased linearly, the calculated *D_syn_* increases with a small periodic deviation from the true value, as shown by the blue curve in Figure 4a. The phase of the cyclic error is set to 0. The integer order of single-wavelength interferometry calculated from *D_syn_* (*N_cal_*) and its true value (*N_true_*) are displayed as pink and green curves, respectively, as shown in Figure 4a. *N_true_* is shifted downward for clarity. The deviation between *N_cal_* and *N_true_* led to discrete points in the comparison between the calculated and true distances, as shown in Figure 4b. These points are measurement errors owing to incorrect integer orders, leading to low measurement accuracy. The ratio between the number of points with the correct integer and all points is 87.35%, which is defined as the probability of retrieving correct integer (labeled as *P*).

Figure 5 shows the calculation results when the phase of cyclic error is π, while the other parameters remain the same. Compared to Figure 4b, less measurement error occurs by changing the phase of the cyclic error. In this case, *P* is 96.76%. This indicates that the performance of distance measurement is improved by adjusting the phase of the cyclic error. 

From Figure 4 and Figure 5, the measurement accuracy is highly deteriorated when the integer is incorrect. The relationship between the measurement accuracy and the accuracy of the integer is summarized in Table 1. When the integer is retrieved correctly (Case 0, probability is *P*), the measurement accuracy is determined by the accuracy of single-wavelength interferometry. When the retrieved integer is one plus the true integer (Case 1, probability is $\left(1-P\right)/2$), the measurement accuracy is the accuracy of single-wavelength interferometry plus the NAR of single-wavelength interferometry. When the retrieved integer is its true integer minus one (Case 2, probability is $\left(1-P\right)/2$), the measurement accuracy is the accuracy of single-wavelength interferometry minus the NAR of single-wavelength interferometry. Given that the accuracy of single-wavelength interferometry is much smaller than the NAR of single-wavelength interferometry, the deviation between the measurement result and true distance is around the NAR of single-wavelength interferometry when the retrieved integer is incorrect. 

**Figure 6:**
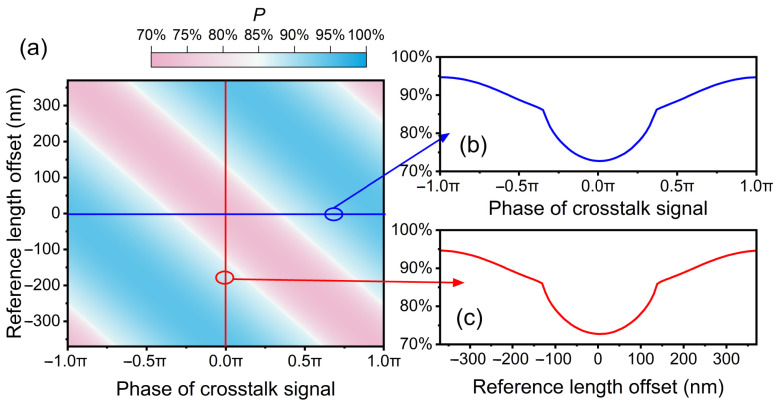
(**a**) The variation of P across different reference length offsets and different phases of crosstalk signal. (**b**) P versus the phase of the crosstalk signal when the reference length offset is 0. (**c**) P versus the reference length offset when the phase of the crosstalk signal is 0.

Because the phase of the cyclic error is determined by the phase of the crosstalk signal and the reference path length, separate investigations into the impact of these two factors on *P* are conducted. In this simulation, the two optical wavelengths are 1540 nm and 1560 nm, respectively, generating a synthetic wavelength of 120.12 µm. The two optical wavelengths are commonly used in many fields and can be easily obtained from a commercial Erbium-doped mode-locked laser. The power of the crosstalk signal is 37 dB lower than that of the heterodyne signal. Figure 6a shows the variation in *P* across different reference length offsets and phases of the crosstalk signal. The reference length is tuned from −*λ*/4 to *λ*/4, corresponding to the phase change of cyclic error from −π to π. At a certain reference length offset and a certain phase of the crosstalk signal, *P* is the probability of retrieving the correct integer when the under-test distance is linearly scanned over half of the synthetic wavelength. As shown in Figure 6b, *P* is plotted against the different phases of the crosstalk signal when the reference length offset is zero. In Figure 6c, *P* is displayed across various reference length offsets, while the reference length offset remains zero. The blue and red curves exhibit the same tendency, indicating that adjusting the reference length offset and the phase of the crosstalk signal have the same influence on *P*. This observation is consistent with Equation (3). Because the phase of the crosstalk signal is an unknown constant during the experiment, the maximum value of *P* can be obtained by adjusting the reference length over a small range.

**Figure 7:**
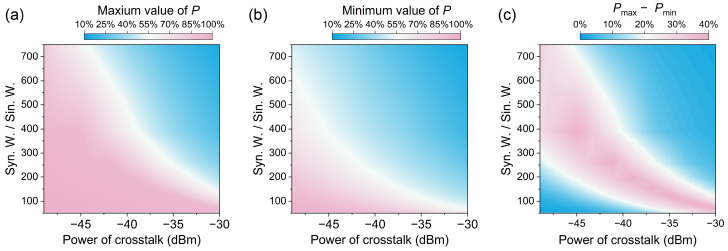
Maximum value (**a**) and minimum value (**b**) of *P* across various synthetic wavelength to single wavelength ratios (Syn. W./Sin. W) and crosstalk power levels. (**c**) The difference between the maximum and minimum values of *P*.

According to Figure 6, *P* varies within a certain range whose upper and lower limits depend on the power of crosstalk and synthetic wavelength to single wavelength ratio. The upper and lower limits of this range are defined as *P_max_* and *P_min_*. *P_max_* and *P_min_* over different synthetic wavelength to single wavelength ratios and different powers of crosstalk are shown in Figure 7a,b, respectively. At each pixel, *P_max_* and *P_min_* are obtained by varying the phase of the cyclic error and scanning the under-test distance by half the synthetic wavelength. Both *P_max_* and *P_min_* deteriorate with an increase in the synthetic wavelength to single wavelength ratio or crosstalk signal intensity. When the crosstalk signal intensity remains constant, an increase in the synthetic wavelength leads to a corresponding increase in the cyclic error due to the magnification factor of 2 × *Λ*/*λ*. Consequently, the error in calculating the integer information of single-wavelength interferometry increases. When the synthetic wavelength to single wavelength ratio is fixed, an increase in the crosstalk signal intensity also leads to a higher probability of obtaining an incorrect integer because of a larger cyclic error. The differences between the *P_max_* and *P_min_* are shown in Figure 7c. The pink region illustrates that the probability of correctly determining integer information can be increased by 30–40% by adjusting the phase of the cyclic error. Consequently, in this region, the performance of distance measurement through combining synthetic wavelength interferometry and single-wavelength interferometry can be significantly improved by fine-tuning the reference length offset. For example, a synthetic wavelength of 302.64 µm is generated by two comb modes with wavelengths of 1552 nm and 1560 nm. The reference path length is 0.388 m, which is 500,000 times 776 nm (NAR of single-wavelength interferometry). Assuming that the phase of the crosstalk signal is 0 and the crosstalk signal is 40 dB weaker than the heterodyne signal in power, *P* is 57.242% when the under-test distance is shifted by multiple times of 776 nm. However, *P* is 93.140% when the reference path length is increased by 388 nm, indicating an increase of 35.898%. This numerical calculation offers valuable insights and recommendations for using appropriate comb mode pairs to generate synthetic wavelengths. It provides recommendations to address the tradeoff between a large NAR and a low risk of failure when combining the two methods, considering the specific power of crosstalk.

## 4. Conclusions

In conclusion, this study provides a comprehensive analysis of the influence of cyclic error on heterodyne-interferometry-based distance measurement using optical frequency comb. In synthetic wavelength interferometry, the cyclic error in the unit of meter is 2 × *Λ*/*λ* times the cyclic error in single-wavelength interferometry, where *Λ* is the synthetic wavelength and *λ* is the optical wavelength. The probability of retrieving incorrect integers of single-wavelength interferometry due to cyclic error in synthetic wavelength interferometry is investigated. This probability is related to the phase and intensity of the crosstalk signal, the length of the reference path, and the ratio between the synthetic wavelength and the single wavelength. The numerical calculation indicates that the influence of incorrect integers on measurement accuracy can be minimized by slightly tuning the reference path length. Choosing an appropriate synthetic wavelength is also important and beneficial for reducing the probability of incorrect integers. These findings contribute to the advancement of precision metrology based on OFC to achieve more accurate and reliable measurements. In addition to distance measurements, this study is applicable to a wide range of applications based on OFC, including surface profiling, imaging, vibration analysis, and so on. 

## Figures and Tables

**Table 1:** The relationship between measurement accuracy and the accuracy of the retrieved integer.

Case	Retrieved Integer–True Integer	Probability	Measurement Result–True Distance
0	0	P	Accuracy of SinWI *
1	+1	(1 − P)/2	≈NAR of SinWI
2	−1	(1 − P)/2	≈−NAR of SinWI

* SinWI: single-wavelength interferometry.

## Data Availability

The data underlying the results presented in this paper are not publicly available at this time but may be obtained from the authors upon reasonable request.
